# Supplementary figures and images for: Extreme restructuring of cis-regulatory regions controlling a deeply conserved plant stem cell regulator
Source: PLoS Genet. 2024 Mar 4;20(3):e1011174. doi: 10.1371/journal.pgen.1011174 (PMC10911594; doi:10.1371/journal.pgen.1011174)

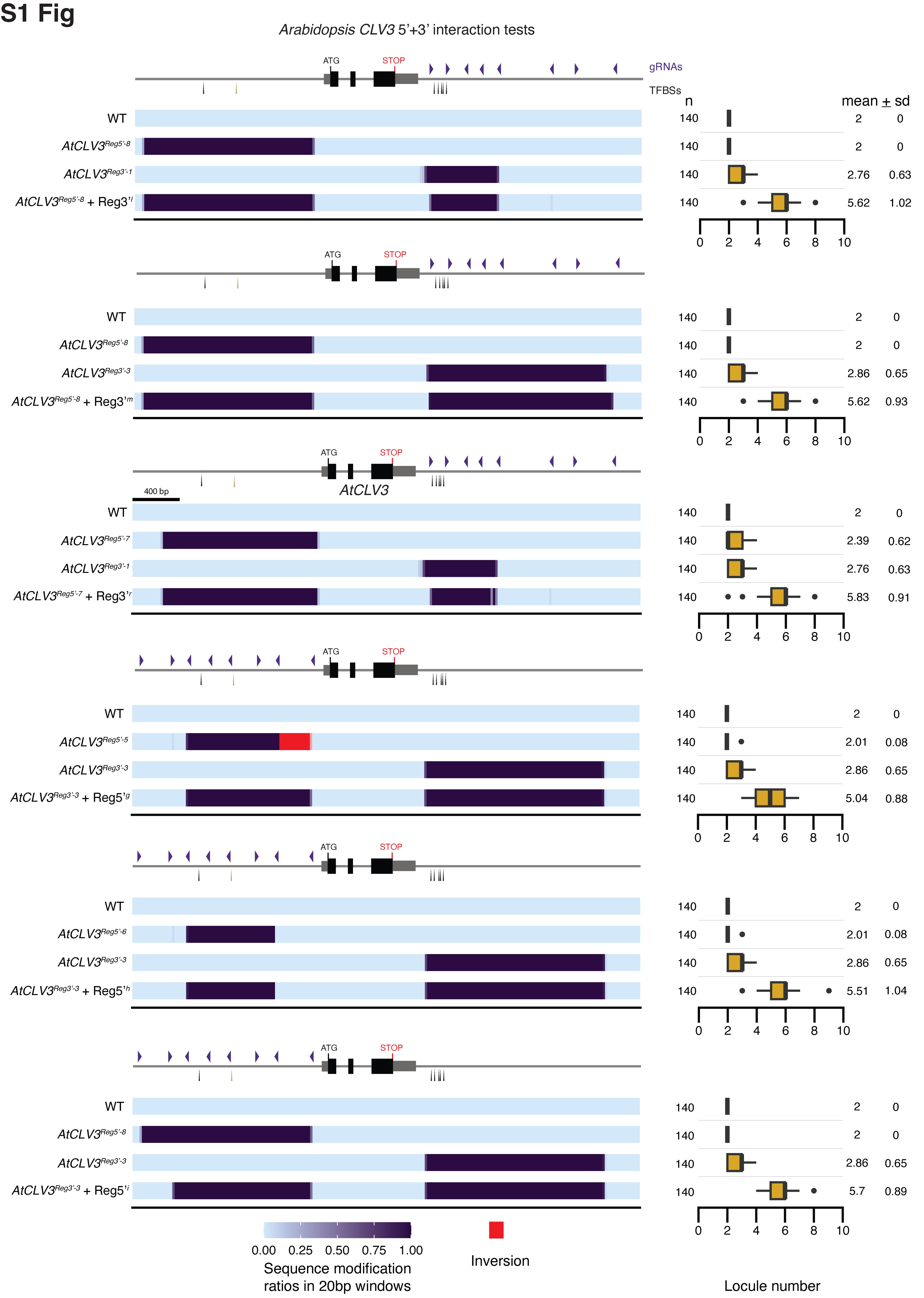

Supplement: S1 Fig — Heatmap representations of the AtCLV3 alleles used in interaction tests, with their locule number quantifications. Each interaction test consisted of a linear model generated from the relationship among four alleles: one 5’+3’ combinatorial allele, one 5’ allele, one 3’ allele, and WT. Purple arrowheads, gRNAs. Black and brown arrows, validated WUS and STM TFBSs. (TIF) [file pgen.1011174.s001.tif]

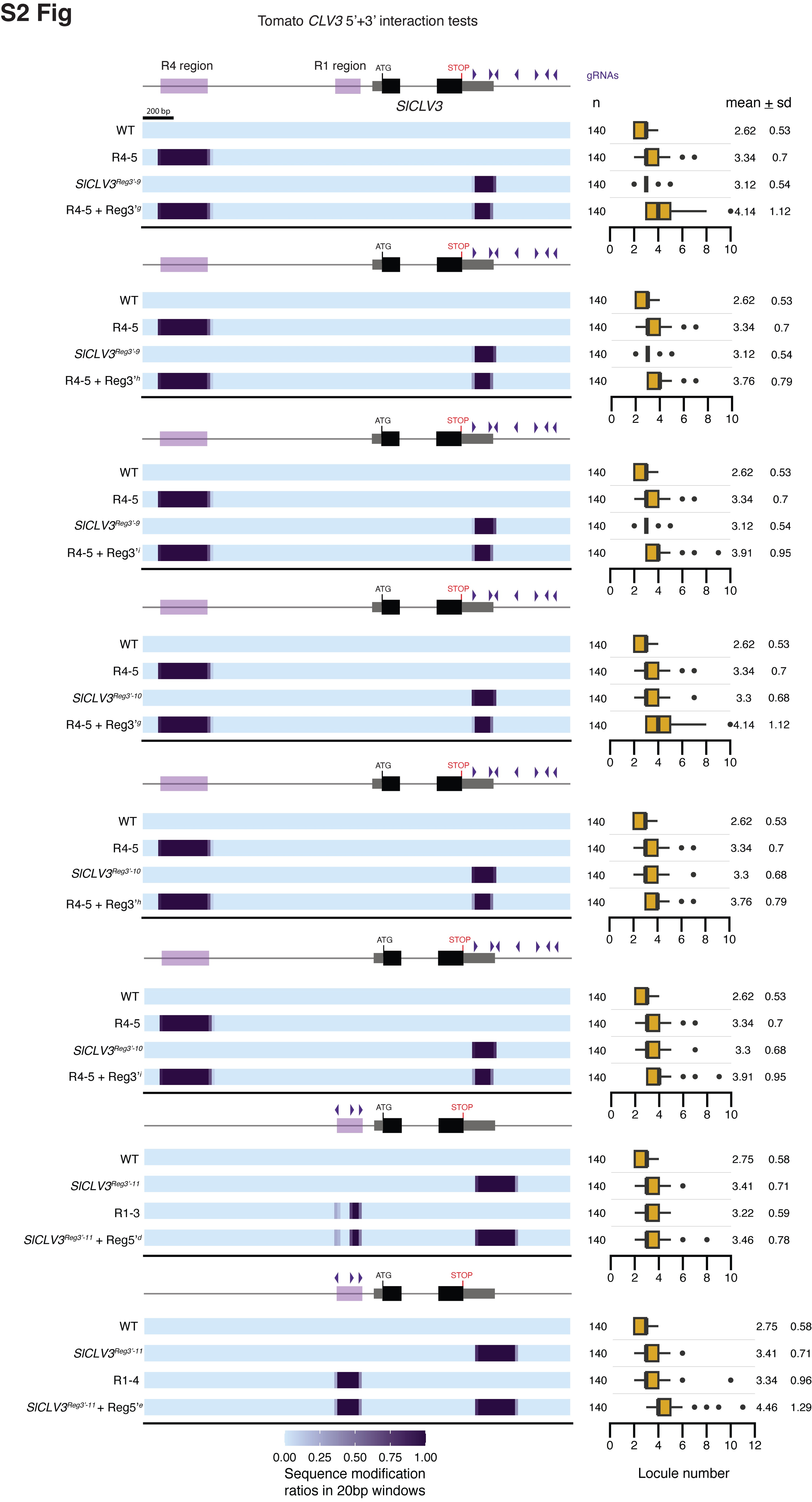

Supplement: S2 Fig — Heatmap representations of the SlCLV3 alleles used in interaction tests, with their locule number quantifications. Each interaction test consisted of a linear model generated from the relationship among four alleles: one 5’+3’ combinatorial allele, one 5’ allele, one 3’ allele, and WT. The R4 and R1 regions previously defined are highlighted by purple boxes on the SlCLV3 5’ non-coding sequence [34]. Purple arrowheads, gRNAs. (TIF) [file pgen.1011174.s002.tif]
