# Supplementary material for: Extreme restructuring of cis-regulatory regions controlling a deeply conserved plant stem cell regulator
Source: PLoS Genet. 2024 Mar 4;20(3):e1011174. doi: 10.1371/journal.pgen.1011174 (PMC10911594; doi:10.1371/journal.pgen.1011174)
Supplement: S1 Table — (PDF) [file pgen.1011174.s003.pdf]

S1 Table. Division of conserved sequences and TFBSs upstream and downstream of *AtCLV3* and *SlCLV3*.

Distribution of Conservatory CNSs

Gene	Region	Total CNS length in region (bp)	Mean length of individual CNS (bp)
AtCLV3	Downstream	237	26.33
AtCLV3	Upstream	284	21.85
SlCLV3	Downstream	101	14.43
SlCLV3	Upstream	254	18.14

Distribution of transcription factor motifs within Conservatory CNSs upstream and downstream of *AtCLV3* and *SlCLV3*

Comparison	Motifs present in *SlCLV3*	Motifs present in both	Motifs present in *AtCLV3*
5'+3' *SlCLV3* vs 5'+3' *AtCLV3*	40	**50**	333
5' *SlCLV3* vs 5' *AtCLV3*	60	24	244
3' *SlCLV3* vs 3' *AtCLV3*	6	1	193
3' *SlCLV3* vs 5' *AtCLV3*	4	3	265
5' *SlCLV3* vs 3' *AtCLV3*	49	35	159

Distribution of 50 shared motifs among upstream and downstream Conservatory sequences of *AtCLV3* and *SlCLV3*

Gene	Shared motifs found 5’	Shared motifs found 3’
AtCLV3	27	36
SlCLV3	46	4

JASPAR Matrix ID of 50 overlapping motifs

Matrix ID
MA1372.1
MA1346.1
MA1823.1
MA0561.1
MA0949.1
MA0967.1
MA1178.2
MA1405.1
MA1040.1
MA1298.1

MA1016.1
MA1047.2
MA1323.1
MA1368.2
MA1280.1
MA0951.1
MA1348.1
MA0565.2
MA1015.1
MA2025.1
MA1750.1
MA1767.1
MA1278.1
MA1070.2
MA1732.1
MA1783.1
MA0588.1
MA1281.1
MA0950.1
MA1324.1
MA1014.1
MA0121.1
MA1737.1
MA0129.1
MA1071.1
MA1036.1
MA1778.1
MA1335.1
MA1336.1
MA1679.1
MA1408.1
MA1406.1
MA1293.1
MA1068.2
MA1269.1
MA1085.2
MA1363.1
MA0020.1
MA1369.1
MA1181.1
