# Supplementary material for: Extreme restructuring of cis-regulatory regions controlling a deeply conserved plant stem cell regulator
Source: PLoS Genet. 2024 Mar 4;20(3):e1011174. doi: 10.1371/journal.pgen.1011174 (PMC10911594; doi:10.1371/journal.pgen.1011174)
Supplement: S2 Table — gRNAs used in Arabidopsis CRISPR, gRNAs used in tomato CRISPR, and genotyping/sequencing primers. (PDF) [file pgen.1011174.s004.pdf]

S2 Table. Oligos used in this study.

gRNAs used in Arabidopsis CRISPR

Name of gRNA	Sequence 5'-3' (including PAM)
***AtCLV3* upstream – proximal 1.5 kb CRISPR construct**	
gRNA1	**ATTTATAGCGTAAGCCTACAAGG**
gRNA2	**AAAGTTGTATAAAACGGCAGGGG**
gRNA3	**TGATATATTAGAGTATGTGCCGG**
gRNA4	**AATAGCATCTAAATATGAGAAGG**
gRNA5	**AATATGGATGATACCTTAATCGG**
gRNA6	**TCTGACACGTGCCCATCCGATGG**
gRNA7	**AAAAAGTAGTGGCACCTTATTGG**
gRNA8	**GATGCAGATCTTTAGCAGTATGG**
***AtCLV3* upstream – proximal and distal 3.8 kb CRISPR construct**	
gRNA1	**TTTGGTAATGAAATGAGAAGGGG**
gRNA2	**TGATATATTAGAGTATGTGCCGG**
gRNA3	**AATATGGATGATACCTTAATCGG**
gRNA4	**GATGCAGATCTTTAGCAGTATGG**
gRNA5	**GTGCAGCTCTCAACTCAAGTAGG**
gRNA6	**TTAGATGTGCATGTACATGTGGG**
gRNA7	**AAGTTGATCTATGGTGAGGGTGG**
gRNA8	**CCATTCATAGCTTATTAAGGCGG**
***AtCLV3* downstream**	
gRNA1	**TCTCCAAAGCAATGTACCGTTGG**
gRNA2	**ACCGACTTTGGGGCAGTGACAGG**
gRNA3	**TAAGGATAATAATTAGCTCTAGG**
gRNA4	**GTTATTTGAGGTGGGAAAAGTGG**
gRNA5	**AAGTCTTGGGATGACATTGGAGG**
gRNA6	**TATTGGTTAGTATAGGTGAATGG**
gRNA7	**TTAGTTTACGTCGACTAATTAGG**
gRNA8	**AGGTAGGTATATTACCCAAACGG**

gRNAs used in tomato CRISPR

Name of gRNA	Sequence 5'-3' (including PAM)
***SlCLV3* upstream**	
gRNA1	**GATATACAACAATGGCTGCATGG**
gRNA2	**GACCTTATCCCCTGCCTTTATGG**

gRNA3	**GAAACACCAAATTATGTTGTAGG**
gRNA4	**GAGATCCATAGTACAGTACTTGG**
gRNA5	**GCAGTAACAAGACAGAGTGACGG**
gRNA6	**GTCCAACAATATATGTTTATCGG**
gRNA7	**GACACCACTCGATTTAAATTTGG**
gRNA8	**GCAATGCAAGTAGCTGCAAAAGG**
***SlCLV3* downstream**	
gRNA1	**TTTAGTAAAGGGTAGTATATGG**
gRNA2	**GCTAGCCAAGTTGGAATATTAGG**
gRNA3	**TCAAAGCTATATACATATCAGGG**
gRNA4	**CTCTTCTCAAAAACGTTCGTTGG**
gRNA5	**GATTGTTAACGAATCAGTTGAGG**
gRNA6	**AACTACAAAGGACTTGCAATAGG**
gRNA7	**TACATAACATACACGTTATAAGG**
**R4**	
gRNA1	**GCAGTAACAAGACAGAGTGACGG**
gRNA2	**GTCCAACAATATATGTTTATCGG**
gRNA3	**ATATGTTATCAATAAAAGATCGG**
gRNA4	**GGACACCTGCCCAACCCAATAGG**
**R1**	
gRNA1	**GATATACAACAATGGCTGCATGG**
gRNA2	**GAAAATAGTTAAGAGGCTTTGG**
gRNA3	**GTATTGCCTCAGCATGTAGAGG**

Genotyping/sequencing primers.

**Name of primer**	**Sequence 5’-3’**
**Arabidopsis**	
AtCLV3-Reg5’-proximal-F	**TCTGATCTAATAAATTGTTGGCC**
AtCLV3-Reg5’-proximal-R	**GTAGCAGAAAACTCTTCGAATC**
AtCLV3-Reg5’-cds-F	**GCTTGCTCCATCATATGTTTG**
AtCLV3-Reg5’-cds-R	**CTGACACTGCCTGTCACTG**
AtCLV3-Reg5’-full-F	**CCGGAACCGAACATAGCAAA**
AtCLV3-Reg5’-full-R	**GTAGCAGAAAACTCTTCGAATC**
AtCLV3-Reg3’-F	**GCTGAAGTGAATGTAAGATACG**
AtCLV3-Reg3’-R	**TGGCGAAGCGGATCATGTAA**
**Tomato**	

SlCLV3-Reg5'-F	**AGAGCCTTCCAATAGCTGGC**
SlCLV3-Reg5'-R	**CTGTTTAGGAGTTTCACAGGAGC**
SlCLV3-Reg3'-F	**CACAATGGTGCTAGTCCTAAG**
SlCLV3-Reg3'-R	**GTGTCTGGATATGTTGAAGATG**
SlCLV3-R4-F	**GAGCTAAGATCGAAAAACCGATC**
SlCLV3-R4-R	**GTAGGATCTGGAGAAAGTTGATG**
SlCLV3-R1-F	**CATAAAGGCAGGGGATAAGGTCTC**
SlCLV3-R1-R	**CTGTTTAGGAGTTTCACAGGAGC**
